# Let Me Take Over: Variable Autonomy for Meaningful Human Control

**DOI:** 10.3389/frai.2021.737072

**Published:** 2021-09-14

**Authors:** Leila Methnani, Andrea Aler Tubella, Virginia Dignum, Andreas Theodorou

**Affiliations:** Department of Computing Science, Umeå University, Umeå, Sweden

**Keywords:** responsible AI, AI ethics, accountability, responsibility, transparency, variable autonomy, human oversight, adjustable autonomy

## Abstract

As Artificial Intelligence (AI) continues to expand its reach, the demand for human control and the development of AI systems that adhere to our legal, ethical, and social values also grows. Many (international and national) institutions have taken steps in this direction and published guidelines for the development and deployment of responsible AI systems. These guidelines, however, rely heavily on high-level statements that provide no clear criteria for system assessment, making the effective control over systems a challenge. “Human oversight” is one of the requirements being put forward as a means to support human autonomy and agency. In this paper, we argue that human presence alone does not meet this requirement and that such a misconception may limit the use of automation where it can otherwise provide so much benefit across industries. We therefore propose the development of systems with variable autonomy—dynamically adjustable levels of autonomy—as a means of ensuring meaningful human control over an artefact by satisfying all three core values commonly advocated in ethical guidelines: accountability, responsibility, and transparency.

## Introduction

As the use of Artificial Intelligence (AI) grows, we continue to see increased societal calls for human control and for an AI development pipeline that follows our legal, ethical, and social values. In particular, many public and governmental organisations have been producing guidelines for the development and deployment of *responsible AI* systems (Jobin et al., 2019). These documents, while providing high-level guidance on the core values that should drive system development and deployment, often provide no explicitness on how to *interpret* and operationalise such values (Theodorou and Dignum, 2020). This focus on the high-level thus provides no single definition of what it means to adhere to these values, making it challenging to first implement and then assess whether systems adhere to the societal criteria set down in those documents.

An example of differing interpretations can be found in the idea of *human oversight,* a prominent theme across guidelines and other initiatives, with an emphasis on respecting and fostering human autonomy and agency. Technical approaches for the inclusion of human oversight over systems, such as *human-in-the-loop* and *human-on-the-loop,* have been much discussed in the academic literature (Amershi et al., 2014), policy documents (European Commission, 2019), and popular science communication (Wang, 2019). However, when it comes to responsible AI, the notion of human oversight extends beyond mere technical human control over a deployed system: it also includes the responsibility that lays in the development and deployment *process*, which entirely consists of human decisions and is therefore part of human control. The concept of *meaningful human control,* developed for the critical area of autonomous weapons, extends beyond mere oversight by including design and governance layers into what it means to have effective control (Horowitz and Scharre, 2015; Santoni de Sio and van den Hoven, 2018; Van der Stappen and Funk., 2021). Meaningful human control refers to control frameworks in which humans, not machines, remain in control of critical decisions, e.g., in the case of autonomous weapons, where the concept was first introduced, humans decide—and bear the responsibility of—when the weapon is allowing the use of lethal force.

In this paper, we argue that the core values of *accountability*, *responsibility* and *transparency* are necessary to ensure meaningful human control in the wider socio-technical sense. Indeed, in this definition, meaningful human control requires taking into consideration the relevant human agents, relevant moral reasons, and appropriate level of responsiveness to those reasons (Santoni de Sio and van den Hoven, 2018). In fact, meaningful human control over a system is not achieved by simply having human presence to authorise the use of force (Santoni de Sio and van den Hoven, 2018; Van der Stappen and Funk., 2021). Rather, it requires the interaction between the user and system to be done in a transparent, traceable manner. If an action is challenged or otherwise requires evaluation, then at least one responsible human along the causal chain can be identified and held accountable. At the same time, the system needs to be developed in a responsible manner by taking into consideration any soft and hard policy and integrating the means for the system to be responsive to the human moral reasons that are relevant to the given circumstance.

Further, we introduce *variable autonomy* to operationalise meaningful human control. Variable autonomy refers to the ability to dynamically adjust the levels of autonomy of the system, i.e., the level of autonomy can switch anywhere between and including full autonomy or complete teleoperation (Chiou et al., 2019). In a system with variable autonomy, the user can take (or relinquish) control over certain (or all) subsystems. As part of our contribution, we argue that in order to effectively design systems that allow for dynamically adjusting the autonomy level we need to consider the same aspects of accountability, responsibility and transparency that constitute meaningful human control. Indeed, by their nature, systems with variable autonomy must include explicit deliberations of the dimensions of autonomy that are afforded, the contexts encountered and the human operator’s knowledge and ability: precisely the considerations that are required to have meaningful human control.

The paper is structured as follows: first, we discuss different approaches to human control in the literature. Next, we discuss how accountability, responsibility and transparency build up to meaningful human control in the socio-technical sense. We then introduce variable autonomy (VA) and show how VA ensures meaningful human control. Finally, we reflect on how VA might look like in current applications of AI and propose some ways forward.

## Human-in/On/Out-of-the-Loop and Human Control

*Human oversight* is a key component in the design of AI systems that support human autonomy and decision-making. This is highlighted by the AI High-Level Expert Group in the European Commission (2019) “Ethics guidelines for Trustworthy AI,” where *human-in-the-loop* (HITL), *human-on-the-loop* (HOTL) and *human-in-command* (HIC) are presented as governance mechanisms that can potentially aid in achieving human oversight. The keyword *loop* may originate from control theory, where the system is engaged in a continuous cycle of measuring and adjusting itself towards achieving a desired state (Norman, 1990). However, in the context of socio-technical systems like the ones discussed in this paper, the idea of “the loop” widens to contain the entire lifecycle of the system, by spanning across all its phases from development to deployment and beyond.

In this section, we discuss established frameworks for the inclusion of humans in the loop. Indeed, the human’s optimal position relative to the loop will vary depending on the human’s role as well as the overall context under which the human and system operate (Grønsund and Aanestad, 2020). Furthermore, we discuss how a static notion of human presence or oversight does not suffice for maintaining human control, and the need for alternative more adaptable solutions exist.

### Human-In-the-Loop

In HITL, the human plays an integral role throughout the entire operation, influencing every decision cycle of the system. This is desirable and often necessary in environments where near optimal performance is required, and machine performance suffers such as those that are dynamic, highly complex or uncertain (Marble et al., 2004; Leeper et al., 2012). For instance, interactive machine learning methods can be used to solve problems for which insufficient data exist or to help deal with complex datasets that capture rare events. The human is brought into the loop during the learning phase of the algorithm to provide indispensable expert knowledge that it cannot acquire on its own (Holzinger, 2016). This model serves as a powerful tool to not only improve system accuracy and efficiency, but also to regulate its behaviour. However, requiring human input at every step in the decision cycle can be inefficient and introduce bottlenecks in the system (van der Stappen and Funk, 2021). Furthermore, the human may not have enough information—or courses of action—to effectively influence the system at every decision (Horowitz and Scharre, 2015). When human involvement is not necessary at every decision step, the HOTL model can be sufficient to regulate system behaviour.

### Human-On-the-Loop

In HOTL, the human steps back during the execution of the operation to assume a supervisory role (Chen and Barnes, 2014), influencing the system by monitoring its behaviours and interjecting only as needed*.* This has many benefits in industrial robotics, for instance, where one human supervisor can oversee multiple assembly robots, checking performance and interrupting only if system failure occurs. In order to intervene, the supervisor must be able to maintain awareness over the system’s status as well as the environment in which it operates. Coordination becomes increasingly difficult to manage as systems grow more complex; especially when multiple agents are involved in the operation (Scerri et al., 2003). Furthermore, if the human does not place realistic trust expectations onto the system, i.e. either over trust and rarely intervene or distrust and intervene too often, the performance of the system will be compromised—potentially even leading to safety concerns (Lee and See, 2004).

### Human-Out-of-the-loop

In situations where humans lack local knowledge, expertise, or timely reaction to optimally respond to the environment, *human-out-of-the-loop* is more appropriate. In these circumstances, autonomy is more of a necessity than a convenience (Castelfranchi and Falcone, 2003). For instance, advanced driver-assistance systems in vehicles promote road safety by detecting and alerting the driver of incoming collision threats and overriding control if necessary. Human error and slow response to time-critical operations motivate the need for full autonomy where the human is pushed entirely out of the control loop, allowing the system to independently execute its task (Kaber and Endsley, 2004). In the case of monotonic systems, human oversight remains even when out of the loop because the behaviours are explicit and known. This is not necessarily the case for AI systems that can deviate from what is expected, e.g., in the case of multi-agent systems, where the human designer cannot influence unexpected emergent behaviour of the organisation (Van der Vecht et al., 2007). HIC addresses this by requiring human involvement in defining conditions for its governance, operation, and use, as well as determining the appropriate evaluation and sanction processes, ensuring human oversight is not lost.

Still, fully autonomous systems cannot always eliminate the risk of human error. The out-of-the-loop performance problem emphasises the issues of skill degradation and reduced situational awareness limiting the human operator’s ability to manually interfere in system operations in case of failure (Endsley and Kiris, 1995). Moreover, autonomous systems can propagate biases learned from human data and can reinforce any systematic discrimination found in society. This point was highlighted in the New York State Department of Financial Services, 2021 “Report on the Apple Card Investigation”, stating that credit scores today perpetuate societal inequality even when calculated in compliance with the law. Striving only for full autonomy can divert attention away from the goal of developing human-centric AI, where human agency is supported and never undermined (Bryson and Theodorou, 2019). This is precisely a reason why human oversight is important.

Choosing one model of human oversight over another is entirely dependent on the context in which the system is deployed, the independent capabilities of the system, the user’s trust in the system (Muir, 1994), as well as the potential risks imposed on society. Indeed, it is often not only a single human who is in the loop, but rather a larger group. Rahwan (2018) uses the term “society-in-the-loop” to refer to the combination of HITL control with a social contract. The challenge thus becomes one of balancing stakeholders’ competing interests and managing issues of coordination. In many real-world applications, the environment is continuously changing and the demand for human or system involvement, i.e., mechanism for oversight, will vary (Marble et al., 2004).

Still, it should be emphasised that human presence is not sufficient for meaningful control from a responsibility standpoint. One may have insufficient information to influence the process rationally, or limited control over parts of the system and no ability to influence other critical components of the causal chain (Horowitz and Scharre, 2015). For meaningful human control, the decision-making system must be able to both *track* relevant moral reasons and *trace* back to an individual along the chain who is aware and accepting of the responsibility (Santoni de Sio and van den Hoven, 2018). Moreover, contexts that change will demand changing levels of responsiveness—a key characteristic of variable autonomy, which is further described in section 4 (Marble et al., 2004).

## Accountability, Responsibility, and Transparency for Meaningful Human Control

Responsible AI rests on three main pillars: *Accountability*, *Responsibility*, and *Transparency* (Dignum, 2019)*.* In this section, we discuss these three values in relation to the human control of our system. First, we discuss why we should—always—be striving for accountability and the importance of identifying the relevant actors along the causal chain of responsibility. Then, how transparency aids in tracing back to said actors who can ultimately be held accountable, and how transparency on its own provides no guarantee of accountability, robustness, or the observation of good practices.

### Accountability and Responsibility

As incidents occur—and sometimes reoccur—the ability to effectively hold the responsible parties answerable for a system’s behaviour is essential for maintaining the public’s trust to the technology (Knowles and Richards, 2021). Yet, multiple scholars have raised concerns over an ongoing *accountability gap*, i.e., current moral and legal frameworks fail to explicitly answer who should be held responsible for the actions taken by an autonomous system and how (Raji et al., 2020; Santoni de Sio and Mecacci, 2021). Although the systems themselves cannot be granted legal personhood and held accountable, the organisations and individuals who may be benefiting through their development, deployment, and use can (Bryson et al., 2017; Solaiman, 2017). Those organisations and individuals are part of a “chain of responsibility” and need to be able to explain and justify their decisions (Dignum, 2017). After all, accountability is the state of being answerable for a system’s actions and its potential impacts (Narayanan 2018). However, the exact scope of the justification given for the actions and impact of a system depends on who is asking for them. Bovens (2007) breaks down accountability into five distinct types, each with its own enforcement mechanisms and means of control over an actor’s behaviour:

1. *legal accountability* is when civil or administrative courts enforce legislation;
2. *professional accountability* is when professional bodies enforce codes-of-conduct and good-design practices;
3. *political accountability* is when elected representatives, e.g., in a parliament, scrutinise—and intervene—to the actions taken of other politicians and political-appointed civil servants;
4. *administrative accountability* is when independent external administrative and financial supervision (e.g., auditing offices) exercise oversight;
5. *social accountability* is when the public or non-governmental organizations hold organisations and individuals accountable for their actions. While direct sanctions are not possible, social responsibility may lead to boycotting and other indirect measures against someone’s actions. Social responsibility is linked to self-regulation activities, as organisations try to maintain their social standing (Jobin et al., 2019).

Each type of accountability can be seen as *a* means of control: it compels, under the “threat” of being held accountable, *responsible actors* to adhere to specified regulations and practices (Bovens, 2007). These responsible actors are all part of a “chain of responsibility,” which includes anyone influencing and influenced by the technologies and policies that are used to develop and govern our systems: from researchers to developers to deployers to users to policymakers (Dignum, 2017). For stakeholders to act, they first need to acknowledge and understand their own responsibilities. Education and governance initiatives, e.g., introducing the need for professional certification, can help make those responsibilities explicit (Theodorou and Dignum, 2020).

It is after all foundational for the effective governance of these systems to recognise that we cannot separate the technology, or the artefact that embeds that technology, from the wider socio-technical system of which it is a component (Mittelstadt and Floridi, 2016; Dignum, 2019). Only then can we increase our ethical and legal foresight and establish practices of accountability and responsibility by looking at technical solutions, socio-organisational activities, as well as processes performed in connection to the technology. Responsibility practices in the socio-organisational level include, for example, the use of software development practices for code robustness, maintainability, and reusability (Bryson and Theodorou, 2019; Raji et al., 2020). At the same time, responsibility as a technical solution includes ensuring technical robustness (Baldoni et al., 2021), the means of linking a system’s decision to its key stakeholders and having the ability for a system to reason for its actions within a specified moral framework (Dignum, 2017).

While responsibility is forward thinking, i.e., acting to deter incidents and violations of our ethical and legal values from occurring, accountability is “a form of backward-looking responsibility and provides an account of events after they occurred” (Van der Stappen and Funk, 2021). To perform its function, accountability requires not only the methods of holding people into account, e.g., legislation in the case of legal accountability, but also the means of tracing actions back to the appropriate responsible party (Van der Stappen and Funk, 2021). Our ability to effectively maintain meaningful human control with accountability relies on having the appropriate auditing and traceability mechanisms in place to track the events that led to a system’s behaviour and which of the stakeholders is responsible for those system actions (Horowitz and Scharre, 2015; Santoni de Sio and van den Hoven, 2018). Approaches such as algorithmic transparency and traceability, which we discuss next, can help us do that.

### Transparency

Transparency is the single most frequently referred to principle in the 84 policy documents reviewed by Jobin et al. (2019), 73 of which promote its need for building socially beneficial AI systems. Yet, transparency can have different interpretations depending on the context in which it is encountered (Winfield et al., 2021). In particular, and where it concerns meaningful human control, transparency can be seen to mean different things:

#### Transparency as Trust

Transparency is often considered as the means of providing an understanding of the emerging behaviour of an agent as it interacts with its environment (Theodorou et al., 2017). The behaviour of systems, alongside with our inherent lack of theory of mind for machines, makes autonomous systems far too complex to effectively debug and understand with “traditional” techniques of testing information systems. User studies have demonstrated how the display of transparency-related information can help users adjust their mental models (Rotsidis et al., 2019; Wortham, 2020) and calibrate their trust to the machine (Dzindolet et al., 2003; Hoff and Bashir, 2015; Mercado et al., 2015). By knowing when to trust—or distrust—the system, the user can make informed decisions on when to accept or reject actions taken by a system and, therefore, exercise more effective control over the system, improving both the safe operation and performance of the human-machine system (Lyons, 2013).

#### Transparency as Verifiability

Others have linked transparency to *traceability*, i.e., the ability to keep a record of information related to a decision (Bryson et al., 2017). Traceability is particularly important for verification and validation (Fisher et al., 2013) and overall testing of a system. Traceability is also fundamental to enabling incident investigators in the identification of the responsible parties (Santoni de Sio and van den Hoven, 2018; Winfield and Jirotka, 2017). However, for the effective attribution of accountability, we need to look not only into the decisions the AI system made, but also into the ones made in its wider socio context. Therefore, auditing frameworks need to look beyond the technical component and instead verify the decisions, policies, and actions taken by all key stakeholders around a system’s lifecycle (Raji et al., 2020).

#### Transparency as Fairness

Transparency is also presented as a means of pursuing fairness in algorithmic decision-making (Jobin et al., 2019). The data that are used to develop learning systems reflect social biases that are perpetuated and amplified with the system’s continued use. Caliskan et al. (2017) demonstrate how machine learning algorithms trained on language corpora acquire harmful historic biases and reinforce them. Certainly, data is not the only source of bias embedded in AI. Design decisions are directed by human moral agency, which cannot be free of bias. Humans use heuristics to form judgements in decision-making, and while these heuristics can be neutral and useful for efficient input processing, they are culturally influenced (Dignum, 2017; Hellström et al., 2020). This presents the risk of formulating harmful biases that are reinforced through practice. Identifying and addressing unwanted biases to ensure fairness requires transparency at every stage of the AI lifecycle.

#### Transparency as Contestability

Still, we should not consider algorithmic transparency as a panacea. In fact, complete algorithmic transparency may not always be possible or desirable to implement due to technical, economic, or social factors ((Ananny and Crawford 2018). Moreover, focusing on algorithmic transparency ignores the fact that AI systems are part of a complex socio-technical ecosystem. Algorithmic transparency without sufficient openness about stakeholder decisions, interests, and overall context, provides not much more than a peephole into a limited part of the whole socio-technical system. Contrary to popular belief, providing transparency—or even explanations—from the system does not mean that we can effectively contest the decisions (Aler Tubella et al., 2020; Lyons et al., 2021). *Contestability,* i.e., the ability to contest decisions, requires looking beyond why a decision was made. Instead, to adequately demonstrate that a contested decision was both *correct and permissible*, we need to investigate the wider context in which the decision was made. Socio-legal factors, e.g., the fairness of the decision, or even actions of other actors and systems need to be taken into consideration. Our right to contest decisions made for us is not only protected by the Regulation (EU), 2016 GDPR, but it should also be considered an important aspect of human control and further motivate systems with variable autonomy.

## Variable Autonomy

The term *variable autonomy* (or *adjustable autonomy*) is frequently seen in the robotics literature to describe human-robot teams in which the level of autonomy (LOA) of the robot varies depending on the context: from complete human operator control to full robot autonomy (Chiou et al., 2019). Variable autonomy (VA) approaches are therefore adopted with the aim to maximise human control without burdening the human operator with an unmanageable amount of detailed operational decisions (Wolf et al., 2013; Chiou et al., 2016). Because of this versatility, VA approaches are for example put forward for exploratory contexts (Dorais et al., 1998; Bradshaw et al., 2003; Valero-Gomez et al., 2011) where conditions are uncertain and broadband connection is unstable, or for controlling multi-robot systems where the operator’s workload is affected by the number of robots under their supervision (Sellner et al., 2006; Wang and Lewis, 2007).

Beyond robotics, VA is also discussed in the context of multi-agent systems (MAS) where interacting autonomous agents participate in the pursuit of a collective organisational goal (Van der Vecht et al., 2007). This type of system requires some coordination (emergent or explicit) between actors. One extreme type of coordination involves fully autonomous agents that generate their own local decisions without any point of control to influence the emergent MAS behaviour. The other extreme is fully controlled coordination which implies a single point of control that explicitly determines and assigns tasks to each actor. In the latter, each agent still carries out their assigned task autonomously, but they do not decide for themselves what actions to perform. With incomplete information about the environment in which the agents are deployed, fully controlled coordination is susceptible to failure and flexibility at the local level is required. This motivates the consideration of VA to dynamically adjust coordination rules, as well as role and interaction definitions within the system.

### Dimensions of Variable Autonomy

Variable autonomy approaches vary in terms of *which aspects of autonomy* are adjusted, *by whom* (human, agent, or both), *how* (continuous or discrete), *why* (pre-emptive or corrective), and *when* (design phase, operation, etc.). On the one hand, autonomy is composed of many facets that can be curtailed (Castelfranchi and Falcone, 2003; Bradshaw et al., 2004): these include the level of permissions (adjusting which actions the system can undertake autonomously), obligations (number of tasks allocated to the system) or capabilities (regulating access to information or to other agents). Thus, a first dimension of enacting variable autonomy involves concretising exactly which aspects of autonomy are in fact variable. On the other hand, the adjustments of the level of autonomy can be performed by the human in what is known as human-initiative, or by either the human or the agent in mixed-initiative approaches (Marble et al., 2003). Specifying *who* has the ability to allocate autonomy determines the level of human involvement at the meta-level of autonomy control and requires forethought on which considerations trigger a possible change in autonomy levels. Indeed, in human-initiative approaches the operator needs to be presented with the relevant information to decide on autonomy adjustment. Additionally, in mixed initiative approaches the system needs to be programmed with the conditions that trigger a change in autonomy level. Deciding who gets to change the level of autonomy and when is therefore a key dimension in VA architectures.

Both the dimension of *which aspects of autonomy* and the dimension of *who gets to adjust it and when* are considered in the literature in terms of their influence on the design and effectiveness of VA systems (Kaber et al., 2001; Castelfranchi and Falcone, 2003; Scerri et al., 2003). When designing for VA, it is necessary to decide where it lies in terms of these two dimensions. This means that the design process explicitly includes considerations on identifying and documenting which aspects of autonomy are adjustable (including system permissions, access to information, etc.) and on the contexts that trigger a change in LOA. In fact, explicit deliberation on system capabilities and human control in different scenarios (ideally taken in conjunction with all stakeholders affected by a system) is precisely what is required for accountability in intelligent system design (Dignum, 2017; Theodorou et al., 2017), making VA an instance of accountability by design.

Furthermore, in both human-led or mixed approaches, the level of autonomy can be adjusted depending on the context. It has been demonstrated that for some models in which the human has the ability to take over and change the LOA at any point during the system’s use, robot performance and use is improved due to the human’s ability to act directly at the error level (Valero-Gomez et al., 2011). In such scenarios it is crucial that the human be aware of where their attention is needed, and of how to quickly tackle the problem when they take over (Sellner et al., 2006). This necessitates transparency, where not only the appropriate quantity of information about the system must be available, but the information must also be delivered at the appropriate time and in the appropriate manner such that it is immediately understood and processed by the relevant human assigned to intervene. Whereas this aspect is a challenge in the implementation of VA, it immediately aligns such systems with the transparency standards increasingly demanded by society such as those outlined in the European Commission (2019) “Ethical Guidelines for Trustworthy AI”.

### Variable Autonomy for Responsible AI

The implementation of VA is often discussed in relation to the operational requirements that ensure one (or many) human operator(s) can maintain control over the system on a technical level (influence over a system to adjust its actions). We argue that these same deliberations, when extended to the wider socio-technical level, give VA an upper hand in terms of accountability, responsibility, and transparency. For a system with VA to be effective, *roles* and *responsibilities* must first be explicitly defined. A role encompasses the set of well-defined tasks that any given entity is expected to independently execute within well-defined conditions of the overall system (Zambonelli et al., 2000). Only by explicitly defining which entities are capable and responsible for which tasks can it be appropriately determined at runtime *who* transfers control of *what* and to *whom*. In order for these entities to adequately fulfil their roles and responsibilities within the system, there must also be an appropriate means for information-exchange such that the current state of the system and state of the environment are well understood. Only by establishing this means of information exchange can the appropriate actor within the system determine *when* a transfer of control is needed and *why*.

#### Variable Autonomy for Accountability and Responsibility

The requirement of making explicit *who* does *what* and *when* extends beyond the roles of human operator and machine. In a socio-technical setting, all key stakeholders who both influence and are influenced by the system should be involved in assigning roles and responsibilities to the relevant actors. Such roles include (but are not limited to) designers, developers, operators, bystanders, and policy makers. With such definitions clearly in place, the value of accountability (a form of backward-looking responsibility) is fulfilled because an account of events and the responsible actors involved can be presented as needed.

#### Variable Autonomy for Transparency

Permissions and access to information is determined by role such that each actor is capable of determining *when* and *where* their action is needed as well as *why* they are required to act. In order to allow for the relevant actor to (re-)gain awareness over the status of system and environment, an account of the relevant events that have occurred must be accessible and available. The system must therefore be inspectable at the appropriate level of abstraction for the relevant entity (an operator and a developer, for instance, will have different views). That is, a means for exchanging just enough information, at the appropriate time, between appropriate actors, in an appropriate manner. With access to information that describes the reasons behind decisions that were made, the system fulfils the value of transparency because the relevant individual is able to gain an understanding as to where their attention is needed and how to appropriately respond.

## Variable Autonomy in Practice: Credit-Scoring System

In this section, we will apply our proposal of VA to the case of the Apple Card, which was under investigation by the New York State Department of Financial Services, 2021 (NYSDFS) after allegations that their credit-lending system, provided to them by Goldman Sachs, discriminated against women (Nasiripour and Natarajan, 2019). We study this use-case to highlight a growing public awareness of companies attempting to hide behind AI to avoid corporate responsibility. First, we describe the events that led to the allegations of gender-bias and the conclusions that were drawn from the NYSDFS investigation. Then, we demonstrate how the requirements of VA outlined in the previous section can address the same issues that triggered the involvement of law enforcement in the first place. We conclude with some considerations about alternative solutions and reflect on how they compare to the VA approach we propose.

The NYSDFS launched their investigation into the Apple and Goldman Sachs after many applicants voiced concerns of gender-bias reflected in decisions made by the Apple Card credit-lending algorithm. This criticism was raised after the system granted male applicants a significantly higher credit limit than their female spouses who, in some cases, had better credit scores. Numerous attempts to appeal resulted in the same response: the decision was made by an algorithm and there was no way to challenge its output. Apple representatives insisted that they do not discriminate, and yet they failed to provide a reasonable explanation for the disparity between credit limits.

While the New York State Department of Financial Services, 2021 did not conclude that Apple and Goldman Sachs exhibited any unlawful discrimination against women, lack of transparency and poor responsiveness to customer appeals were implicated. The Department emphasised that these two features are of particular importance when customer insight into the basis for their credit terms is little to none. Apple and Goldman Sachs failed to provide meaningful control over the situation, as the deployed system did not allow for the ability to track moral reasons for the outcome or the ability to trace back to a responsible individual who could both understand the outcome and explain it to the contesting party in a timely manner. Apple’s policy at the time required the applicants to wait 6 months before appealing the decision made by the system. Only after authorities intervened did the relevant actors present reasons for each individual outcome. If applicants were able to contest the decisions effectively, e.g., speak with a representative who could explain the outcome instead of being told a “computer said so”, then the investigation might have been avoided. We argue that variable autonomy applied to such a case would demand transparency by design, ensuring that the relevant actors can intervene at the right moment and respond appropriately to the contesting individual, thus providing meaningful control over the system.

The first ailment of the Apple credit card programme was lack of transparency. An effective VA approach requires transparency such that all actors along the causal chain are known, and their responsibilities made explicit prior to deployment of the system. Additionally, each actor must be able to obtain an adequate understanding of where their action is required if they are to fulfil their roles and responsibilities. This necessitates appropriate access to the information that is of particular relevance to each individual actor’s role. Apple and Goldman Sachs’ system presented the applicant with insufficient explanations for the decisions that were made. Moreover, Apple representatives could not provide reason beyond “the algorithm said so” because they had no insight into the system that Goldman Sachs supplied to them, i.e., the system was a “black box”. It is argued, however, that Apple accepted a role and responsibility when they launched the credit card programme. Without sufficient information about the system that the programme’s success heavily relied on, they could not fulfil their responsibilities or maintain control.

The second ailment was poor contestability. VA’s requirement of explicitly defining the roles and responsibilities of all actors along the causal chain primes the system for presenting an account of occurrences as needed. We propose the need for a more robust design approach for which Apple representatives are given appropriate role assignments within the wider socio-technical system that match the responsibilities they possess. This way, the individual who is tasked with inspecting the system at the appropriate level (there might be multiple levels of abstraction) and accounting for the decision steps that led to any given outcome would be known. This individual can respond as needed to the contested decision and the involvement of law enforcements could have been avoided.

If the decision was found truly to be biased, then steps can be taken towards amending the fault within the system. With VA, humans can assume control, understand the state in which the system errs, and make a more informed decision for the contesting applicant as well as all other applicants using the same system. A discriminatory system is a failed system and control must be transferred to an entity that can be challenged and held to account.

Other solutions to cases where the opaque system is suspected to be biased include the use of debiasing techniques and conformance testing. If the dataset used by credit lenders is suspected to be imbalanced, then one solution is to re-train the system on a more representative dataset (Noor, 2020). However, careful methods of data collection and bias-testing in pre-processing stages cannot necessarily account for all cases, so there should be robust mechanisms in place to handle the potential for failure. Moreover, data collection methods can be time consuming and expensive, especially if they are to be performed at every occurrence of a detected fault. Representative data cannot ensure a bias-free model, however, especially from a credit-scoring perspective (Hassani, 2020). Historic social biases can be reflected in the data and reinforced by their use in credit score calculations. Constraints can be applied to the model itself in attempts to correct for bias, but it is difficult to ensure fairness across all categories without compromising performance (Kleinberg et al., 2016; Hassani, 2020). Another solution is to perform regular conformance testing such as scheduled audits to the system. While this is useful for accountability (Raji et al., 2020), it is also a time and labour-intensive task that requires major efforts from both parties and cannot always take place.

Still, only throwing more data at the problem or auditing the system periodically solves none of the issues that triggered the investigation into Apple card in the first place: lack of transparency and poor response to appeals. More robust governance mechanisms need to be in place prior to system deployment. Human-in/on-the-loop are governance mechanisms that are inefficient in this case because it is labour intensive and requires humans to continuously oversee the system—it is simply not feasible. The human-out-of-the-loop model provides faster decision-making at a cheaper cost for financial institutions, but it is high-risk in uncertain situations. Therefore, a dynamic solution is more reasonable. Time investments can be made in the training of all actors within the system to inform them of their role and responsibilities and ensure they are fit to serve. With responsibilities specified, each human knows their position along the chain, what parts of the system they can access, what they cannot, and who they need to contact in case an intervention is required, e.g., an appeal. The appropriate actor can be traced along the chain and localise the issue, providing reason for outcome and satisfying meaningful control. The VA solution is versatile and encompasses the values of accountability, responsibility, and transparency. By adhering to these values, Apple and Goldman Sachs would have maintained meaningful control over the system. However, the development of such a VA system is not without open challenges, and the need for careful considerations throughout its design, implementation, and use exists.

## Reflections and Additional Considerations

Determining whether, when and why control should be transferred to other entities are fundamental questions to consider in the development of systems with VA. The answers to these questions are contextual and will vary between systems. It will also depend on the values of the stakeholders involved in the design. Moreover, successful coordination between actors is heavily dependent on both internal (inner workings of the system) and external (environmental) factors that influence overall system stability. For a system with VA to be effective, decisions that require human (or machine) input must first be identifiable. Then, the appropriate entity to transfer control of these decisions to must be capable, available, and authorised to make these decisions without incurring significant costs to the system due to e.g., decision-making delays or miscoordination (Scerri et al., 2003).

In high-risk situations, the assumption that the human will be capable of taking over control immediately without disruption can result in severe miscoordination and ultimate system failure. It is therefore important for the system to consider that the human is not guaranteed to respond to a request for input (Scerri et al., 2003). In other low-risk situations, user neglect is more tolerable, particularly when the alternative is disaster. Neglect tolerance is therefore an important consideration for the design and development of VA transfer of control mechanisms. Allowing for agents to reason about decision uncertainty, costs, and constraints is one way to optimise this transfer of control problem (Scerri et al., 2003). Other hybrid approaches combine logic reasoning with machine learning methods to solve the same. In such systems, the authority to transfer control need not only be reserved for the human but can also be mixed initiative. A transfer of control that is triggered by the system is desirable in circumstances e.g., where the human is not responsive, under extreme stress or in a suppressed cognitive state (Parasuraman et al., 1999).

The human’s cognitive state is another important consideration in the development of VA systems. How situational awareness can be achieved and maintained warrants further research, as the form and the means of presenting information is a system-specific consideration. Cues from safety-critical systems, for instance, might vary in amount of information depending on the situation. This is critical for the avoidance of infobesity, i.e., overload of the humans’ cognitive abilities, and risks having the opposite effect on situational awareness (Endsley and Kiris, 1995). Finally, human factors research in trust demonstrates the need for transparency to enable calibration of trust, otherwise the human may misplace their trust in the system, resulting in the system’s *misuse* or *disuse* (Lee and See, 2004).

## Conclusions and Future Work

As the deployment of AI systems continues to expand across industries, it is becoming increasingly important to ensure that control over any intelligent system is maintained in a way that is both meaningful and practical to its use. In this paper, we described the challenges in maintaining human oversight using governance mechanisms such as human-in-the-loop and human-on-the-loop. We argue that these mechanisms for control do not suffice for the maintenance of what is understood to be meaningful human control as they do not necessarily encompass the requirements of tracking moral reasoning and tracing accountable individuals along the causal chain of responsibility. Moreover, dynamic contexts will demand systems with adaptable levels of human responsiveness. We further discussed the importance of effective governance over intelligent systems by highlighting accountability, responsibility, and transparency as the three main pillars of the responsible and trustworthy development and use of AI.

We presented the concept of variable autonomy as a means of ensuring the effective governance and subsequent alignment of systems with our socio-ethical legal values. We introduced design and implementation considerations needed. For example, the importance of clearly defining the roles and responsibilities of all actors along the causal chain of the system (from designer to end-user), such that all actors are aware of the set of tasks they are responsible for, and the circumstances under which they must execute said tasks*.* This necessitates a means of information availability and exchange between relevant actors such that they are enabled to fulfil their assigned roles. Such are the requirements for VA systems to adhere to the values of accountability, responsibility, and transparency, which in turn ensure meaningful human control.

Moving forward, we intend to apply a quantitative analysis of VA systems for meaningful control. Further study is needed in determining the optimal action selection sequence for transfer of control given uncertainties, costs and constraints imposed on the system. In particular, we are interested in investigating the use of hybrid systems with VA, combining logic reasoning with machine learning methods to optimise this transfer of control problem. We will investigate these combined methods not only to determine *who* to transfer control to and *when*, but also in what manner. These are a few of the questions that we aspire to answer as a step towards determining how best to integrate VA into systems at large, encouraging their responsible development and deployment across all industries.

## Data Availability

The original contributions presented in the study are included in the article/Supplementary Material, further inquiries can be directed to the corresponding author.
